# A functional mechanism for a non-coding variant near *AGTR2* associated with risk for preterm birth

**DOI:** 10.1186/s12916-023-02973-w

**Published:** 2023-07-17

**Authors:** Li Wang, Robert M. Rossi, Xiaoting Chen, Jing Chen, Jilian Runyon, Mehak Chawla, Daniel Miller, Carmy Forney, Arthur Lynch, Xuzhe Zhang, Fansheng Kong, Bo Jacobsson, Leah C. Kottyan, Matthew T. Weirauch, Ge Zhang, Louis J. Muglia

**Affiliations:** 1 Center for Prevention of Preterm Birth, Perinatal Institute, Cincinnati Children’s Hospital Medical Center, Cincinnati, OH USA; 2 Division of Human Genetics, Cincinnati Children’s Hospital Medical Center, Cincinnati, OH USA; 3 March of Dimes Prematurity Research Center Ohio Collaborative, Cincinnati, OH USA; 4 Present Address: Department of Biology, Xavier University, OH Cincinnati, USA; 5 Department of Obstetrics and Gynecology, Division of Maternal-Fetal Medicine, University of Cincinnati College of Medicine, Cincinnati, OH USA; 6 Center for Autoimmune Genomics and Etiology, Cincinnati Children’s Hospital Medical Center, Cincinnati, OH USA; 7 Department of Pediatrics, University of Cincinnati College of Medicine, Cincinnati, OH USA; 8 Divisions of Biomedical Informatics and Developmental Biology, Cincinnati Children’s Hospital Medical Center, Cincinnati, OH USA; 9 Department of Obstetrics and Gynecology, Institute of Clinical Sciences, Sahlgrenska Academy, University of Gothenburg, Gothenburg, Sweden; 10 Department of Obstetrics and Gynecology, Region Västra Götaland, Sahlgrenska University Hospital, Gothenburg, Sweden; 11 Department of Genetics and Bioinformatics, Domain of Health Data and Digitalisation, Institute of Public Health, Oslo, Norway

**Keywords:** Functional studies, Preterm birth, Non-coding variant, AGTR2

## Abstract

**Background:**

Preterm birth (PTB), defined as delivery before 37 gestational weeks, imposes significant public health burdens. A recent maternal genome-wide association study of spontaneous PTB identified a noncoding locus near the angiotensin II receptor type 2 (*AGTR2*) gene. Genotype-Tissue Expression data revealed that alleles associated with decreased *AGTR2* expression in the uterus were linked to an increased risk of PTB and shortened gestational duration. We hypothesized that a causative variant in this locus modifies *AGTR2* expression by altering transcription factor (TF) binding.

**Methods:**

To investigate this hypothesis, we performed bioinformatics analyses and functional characterizations at the implicated locus. Potential causal single nucleotide polymorphisms (SNPs) were prioritized, and allele-dependent binding of TFs was predicted. Reporter assays were employed to assess the enhancer activity of the top PTB-associated non-coding variant, rs7889204, and its impact on TF binding.

**Results:**

Our analyses revealed that rs7889204, a top PTB-associated non-coding genetic variant is one of the strongest eQTLs for the *AGTR2* gene in uterine tissue samples. We observed differential binding of CEBPB (CCAAT enhancer binding protein beta) and HOXA10 (homeobox A10) to the alleles of rs7889204. Reporter assays demonstrated decreased enhancer activity for the rs7889204 risk “C” allele.

**Conclusion:**

Collectively, these results demonstrate that decreased *AGTR2* expression caused by reduced transcription factor binding increases the risk for PTB and suggest that enhancing *AGTR2* activity may be a preventative measure in reducing PTB risk.

**Supplementary Information:**

The online version contains supplementary material available at 10.1186/s12916-023-02973-w.

## Background

Preterm birth (< 37 completed gestational weeks) complicates approximately 10% of pregnancies in the USA [1]. Globally, preterm birth and its complications are the leading cause of mortality in children under 5 years of age [2]. Previous studies have demonstrated that there is a strong genetic component involved in gestational duration and preterm birth [3], though early studies failed to prove statistically significant correlations [4, 5]. These early studies either focused on a set of pre-selected genetic variants or target genes and/or were performed in small cohorts. Advances in genome-wide association studies (GWAS) in the past two decades make it possible to evaluate genetic variants associated with pregnancy complications at the whole genome level in large cohorts of usually more than 5000, providing powerful and robust associations [6, 7]. Our group has recently reported the first 6 genetic loci robustly associated and replicated for altering the risk of preterm birth in the human population [8]. In this study, we examine the *AGTR2* (angiotensin II receptor, type 2) locus, a locus on the X chromosome strongly associated with not only gestational duration but also preterm birth risk as a dichotomous trait [8].

The *AGTR2* locus is particularly compelling in that it has clear developmental roles in maternal physiology and is a potential druggable therapeutic target. *AGTR2* protein has been implicated in a multitude of functions involving vasodilation, cell apoptosis, growth inhibition, natriuresis, nitric oxide production, and collagen synthesis [9–11]. Cell signaling through *AGTR2* is poorly understood, but likely occurs through activation of protein phosphatases, activation of cGMP, and release of phospholipase A2 and arachidonic acid [12]. *AGTR2* is expressed in the developing fetus and then rapidly decreases after birth in most tissues. It is predominantly expressed in the lung, uterus, and endometrium [13, 14]. Function of this gene in reproduction has been suggested in early placental development [15] and implantation or decidualization [16]. As the starting sample materials for both studies were either placental/decidual [15] or whole endometrial biopsies [16], the specific tissue/cell type of action has not yet been identified.

From our GWAS study, the index SNP lies more than 200 kb upstream of the *AGTR2* gene and acts as an eQTL for the *AGTR2* gene [8], suggesting that distal enhancer activity may be involved in the molecular mechanisms underlying *AGTR2* gene expression. However, any variants in the linkage disequilibrium (LD) block with this index SNP can potentially be responsible for the detected association, whereas only a few of them are likely truly causal and functional [17, 18]. Functional studies can provide mechanistic insights by determining whether the distal acting non-coding variants disrupt the DNA binding of transcription factors, chromatin regulators, and/or nucleosomes in an allele-dependent manner [19]. It is also known that molecular mechanisms of action via non-coding genetic variants are highly cell- and tissue-specific [20–23]. To address these issues, we take advantage of recent advances in genomics, epigenomics, and transcriptomics that annotate the diverse gene regulatory elements [24–28]. These studies are often large scale, covering multiple tissues and/or cell types, and include multiple layers of genomic and epigenomic regulatory mechanisms. Mining of such data sets can provide useful insights for causal SNP prioritization and identification and identify potential regulators such as transcription factors and target tissue/cell types in which they operate.

Once a potential causal SNP and target gene have been identified in a relevant tissue/cell type, it is equally crucial to experimentally validate and further elucidate the mechanisms leading to human diseases. As not all human diseases can be recapitulated in vitro, a common approach to demonstrate the functionality of a particular non-coding variant is to (1) identify TFs with altered DNA binding activity via electromobility shift assays (EMSA) or DNA affinity precipitation assays (DAPA) [29]; and (2) create a construct harboring both alleles of the variant and test its ability to alter reporter gene expression [30].

Due to the complexity and uncertainty of the function of the *AGTR2* gene, our study focuses on demonstrating the enhancer activity of the identified non-coding region in regulating *AGTR2* expression, identifying the regulatory proteins involved in allelic regulation, and exploring the potential tissue/cell types in which this regulatory module operates. To this end, we sought to determine the likely causative variant in the noncoding region adjacent to *AGTR2* and to determine the mechanistic effect of this variant on the regulation of *AGTR2* expression. We conducted bioinformatic analysis using publicly available multi-omics datasets. This approach prioritized a single non-coding SNP, rs7889204, to be the most likely causal SNP. rs7889204 is a strong eQTL for the *AGTR2* gene, especially in uterine tissue, with the major “T” (non-risk) allele associated with higher *AGTR2* expression, and the minor “C” (risk) allele with lower *AGTR2* expression. Our method also identified transcription factors with predicted allele-dependent binding at this SNP. Using EMSA, we validate that rs7889204 alters the binding of the CEBPB and HOXA10 TFs, both of which display stronger binding to the T non-risk allele compared to the C risk allele. Reporter assays demonstrate decreased enhancer activity for the risk allele at rs7889204. Collectively, these results support the potential utility of modulation of *AGTR2* activity and/or its CEBPB and HOXA10 regulators for PTB prevention and therapy.

## Methods

### Cell line, culture, and induced decidualization

The cell line used in EMSA and luciferase assay was an immortalized human endometrium stromal fibroblast cell line (T HESC, Mor lab, Yale University, corresponding to ATCC CRL-4003). Culture and further decidualization conditions are described in [31] with minor modifications. Specifically, the decidualization medium is supplemented with 2% FBS, 0.5 mM cAMP(Sigma 23583–48-4), 1uM MPA (Sigma, M1629), 1*Pen/Strep and 0.24 g NaHCO_3_ per 200 mL DMEM medium.

### Computational analysis to predict and prioritize functional SNPs

We performed linkage disequilibrium (LD) expansion using the PLINK software package (version 1.90b) with EU ancestry (LD *r*^2^ > 0.8). This resulted in a total of 49 candidate SNPs. We then annotated these SNPs with eQTL data. We collected functional genomic datasets including ENCODE [28], Cistrome [32], PAZAR [33], Re-Map [34], and NIH Roadmap Epigenomics [35]. All datasets were indexed by their genomic coordinates, which were used to intersect with the LD expanded list of SNPs of interest. To help prioritize functional SNPs, SNPs with overlapping TF ChIP-seq signals were scanned for the putative binding signals using TF binding models based on protein binding microarray data using the Cis-BP database. After cross reference of the above results, we identified rs7889204 as the candidate causal SNP and FOXA1, CEBPB, and HOXA10 as transcription factors with predicted differential allelic binding at this T/C SNP.

### Genotype-Tissue Expression quantitative trait (eQTL) analysis

We examined the tissue-specific expression of *AGTR2* using the Genotype-Tissue Expression (GTEx) Portal (https://gtexportal.org/). Significant single-tissue eQTLs for *AGTR2* in all tissues were extracted from GTEx Analysis Release V8. The distribution of the eQTLs and the pairwise LD pattern were visually inspected using the GTEx Locus Browser (Fig. 2A). Single-tissue eQTLs for rs7889204 were extracted and examined using eQTL violin plot (Fig. 2B).

### 3D Genome Interaction Analysis using 3DIV

We used rs7889204 as the bait on the HiC page under the interaction visualization tab on the 3D Genome Interactive Viewer and Database [36] (3DIV) (http://kobic.kr/3div/). We followed the tutorial using default settings with the exception that we set the threshold on distance normalized interaction frequency to be 2.00. We started with a selection of all possible data sets and then ranked them by gene name. Chromatin looping interaction signal was found between this SNP and the promoter region of *AGTR2* in a fibroblast cell context (Fig. 2C).

### Electrophoretic mobility shift assay

Electrophoretic mobility shift assays (EMSAs) were performed as previously described [29, 30] to determine whether the candidate variant at the *AGTR2* locus differentially affected the binding of the predicted transcription factors, namely Forkhead box protein A1 (FOXA1), CCAAT/enhancer-binding protein beta (CEBPB) and Homeobox protein A10 (HOXA10). Briefly, we prepared nuclear protein extracts as previously described with minor modifications [29]. Decidualized hESF cells were trypsinized, washed twice with cold PBS, counted, resuspended in PBS as 10^7^ cells/ml, then centrifuged 3300 g for 2 min, 4 °C. PBS was removed, and 10^7^ cells were resuspended in 400 μl CE buffer (10 mM HEPES (pH7.9), 10 mM KCl, 0.1 mM EDTA, 1 mM dithiothreitol (DTT), and 1X HALT protease/phosphatase inhibitor (ThermoFisher)), then incubated on ice for 15 min. Cells were mixed with 25 μl of 10% Nonidet P-40 by pipetting, and then nuclei were pelleted at maximum speed for 3 min, 4 °C. Nuclei were resuspended in 30 μl NE buffer (20 mM HEPES (pH7.9), 0.4 M NaCl, 1 mM EDTA, 1 mM DTT, 1X protease/phosphatase inhibitor) by vortexing, incubated on ice for 10 min, then centrifuged 3300 g for 2 min, 4 °C. Supernatants (nuclear proteins) were removed from pelleted debris and stored in small aliquots at − 80 °C. Protein concentration was determined by BCA protein assay (ThermoFisher). SNP rs7889204 was used to prepare 5′ IMR700 dye–labeled 35-bp duplexed oligonucleotide probes (Integrated DNA Technologies). SNP oligonucleotide sequences were obtained from the National Center for Biotechnology Information dbSNP database (https://www.ncbi.nlm.nih.gov/snp) by using human genome build 37. The sequence of probes are as follows:

- reference “T” allele is:
  - /5IRD700/AA CCC AAA AGC AAA TGC AAT AAA AAC AAA GAT AAA TAG C
- non-reference “C” allele oligo sequence is:
  - /5IRD700/AA CCC AAA AGC AAA TGC AAC AAA AAC AAA GAT AAA TAG C

EMSA binding reactions contained 6 μg of nuclear extract, 100 fmol of the labeled probe, 2μL of 10 × binding buffer, 1 μg of Poly(dI-dC), 1μL of 40% glycerol, 2μL of 25 mmol/L dithiothreitol/2.5% Tween 20, 1μL of 1% NP-40, 1μL of 100 mM MgCl_2_, and ultrapure water (LI-COR Biosciences) in a total reaction volume of 20μL. Products were electrophoresed on native 5% Tris–Borate-EDTA precast polyacrylamide gels (Bio-Rad, Life Sciences, Hercules, Calif). Gels were prerun at 70 V/cm for 30 min in 0.5 × nondenaturing Tris–Borate-EDTA buffer, and samples were loaded, run for 60 min, and then imaged with an Odyssey CLx imager (LI-COR Biosciences). The hESF (human endometrium stromal fibroblast cell) cell extracts were supplemented with purified TF protein as above, as endogenous TF protein was not initially detected on EMSA. All candidate TF proteins examined were generated from an overexpression vector after nuclear lysate purification and tagging with a DDK motif (Origene). 50fmol fluorescent oligo DNAs were then added to the appropriate protein/binding mix and incubated for 20 min at room temp. For supershift assays, anti-DDK antibody (Origine #TA50011-100) was incubated with the nuclear lysate/binding buffer for 20 min prior to the addition and incubation with oligo DNA. 1 × orange loading dye (LI-COR kit) was added to samples, which were then resolved on (pre-cast, pre-run at 100 V for 60 min) 6% TBE gels (Novex, ThermoFisher) in 0.5 × TBE buffer for 80 min at 80 V (4 °C). Fluorescent bands were then imaged using a LI-COR chemiluminescent imaging system. EMSA experiments display representative panels of 2–3 replicates.

### Luciferase assays

Luciferase reporter constructs were cloned by using the pNL1.1 nanoluciferase reporter vector (Promega, Madison, WI) as a backbone. For the *AGTR2* promoter reporter construct, PCR primers were used to amplify a 1 kb sequence including the first 816 base pairs upstream of the transcription start site, and the region was subsequently cloned into the pNL1.1 backbone. For the putative *AGTR2* regulatory GWAS locus upstream of the *AGTR2* promoter, two complementary oligonucleotides were designed to create fragments for insertion into this backbone. The GWAS regulatory region was comprised of a 4.3-kb region surrounding rs7889204 (which includes three other SNPs(dbSNP_153), rs4478734 (which is an eQTL for *AGTR2* in uterus and adrenal glands but not predicted to alter TF binding), rs9724136(no significant eQTL data in any tissue) and rs2310841(no entry in GTEx portal, not predicted to alter TF binding). The region was inserted into the pNL1.1 nanoluciferase reporter construct in front of the *AGTR2* promoter. Site-directed mutagenesis was performed to generate a construct with all three non-reference SNPs and a construct with the non-reference (risk) allele of rs7889204 using the GeneArt Site-Directed Mutagenesis System (Invitrogen, Darmstadt, Germany). Sequences were confirmed by Sanger sequencing.

250 ng of nanoluciferase experimental constructs were transiently co-transfected with 250 ng of pGL3-control firefly luciferase reporter plasmid into endometrial fibroblast cells grown to ~ 90% confluence using Lipofectamine 3000 (L3000015, Thermo Fisher Scientific) in a 48-well cell culture plate using 500 ng of total plasmid per well. For decidualization, after 24 h, transfected cells were treated with cAMP, progesterone, and estradiol for 48 h as we have previously reported. Then cells were lysed and both nano and firefly luciferase activities were measured in the same sample by using the Nano-Glo® Luciferase Assay System (Promega, Madison, WI). The nanoluciferase measurement for each well was divided by the firefly luciferase measurement in order to account for cytotoxicity, small differences in cell number, and transfection efficiency. Each well was further normalized to the pNL1.1 nanoluciferase reporter backbone to control for baseline activity of the vector. Three independent experiments were performed, and three replicate samples were assayed in each experiment. Student’s *t*-test was performed to determine significant differences between the risk and non-risk alleles for rs7889204.

## Results

### Prioritization of functional SNPs and predication of allele-dependent TF binding

**Fig. 1:**
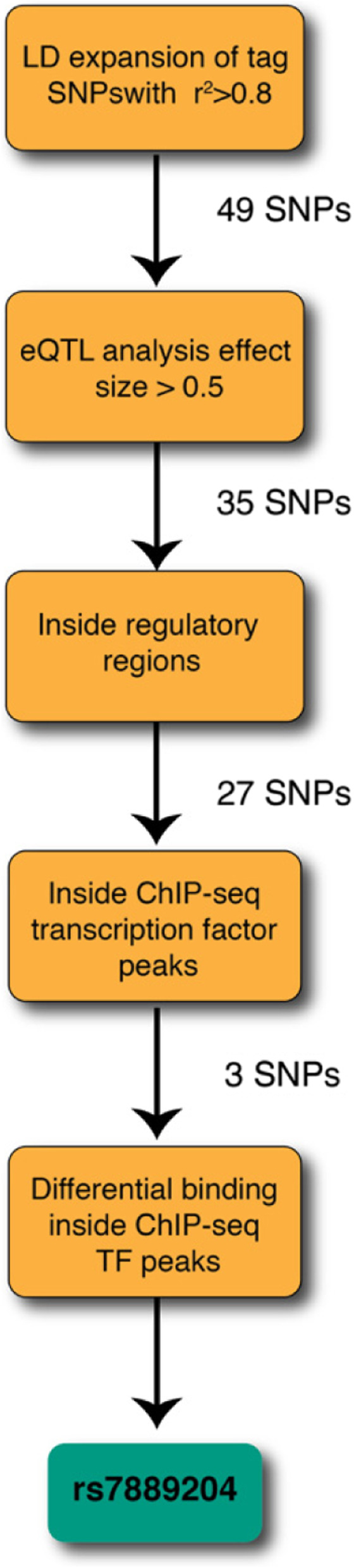
Strategic approach for SNP prioritization and transcription factor prediction. Our procedure started with an LD expansion of the GWAS-identified index SNP from the discovery phase using criteria *r*^2^ > 0.8 using the PLINK software package (version 1.90b) with EU ancestry. The resulting set of 49 SNPs was used for subsequent analysis to prioritize the causal SNP and to predict the differential binding of transcription factors. This analysis identified rs7889204 as the most likely causal SNP

The *AGTR2* adjacent genetic association locus falls within a non-coding region of the human genome on chromosome X, which suggests that one or more of these variants might functionally act as enhancers by altering gene regulatory mechanisms. Although *AGTR2* was the closest gene to this identified region and biologically plausible as the consequential gene affecting preterm birth as previously reviewed, the index SNP from the discovery phase was located over 200 kb from the *AGTR2* transcription start site (TSS). As such, we first determined whether this GWAS locus interacts with the gene *AGTR2* given the substantial distance between regions. It is promising to know that the locus falls in the same TAD (topologically associated chromatin domains) domain with the *AGTR2* gene in human ESC (embryonic stem cells) and IMR90 cell lines [37]. TAD domains are known to be conserved among cell types and even different species. They are the unit of genome organization that allow for coordination of gene regulation programs and integration of transcriptional signals [38]. Next, a total of 49 SNPs were identified as close proxies in linkage disequilibrium (LD) with the index SNP from the discovery phase using criteria *r*^2^ > 0.8 using the PLINK software package (version 1.90b) [39] with European (EU) ancestry (Fig. 1 and Additional file 1, spreadsheet SNPs in LD). Out of these 49 SNPs, 35 are expression quantitative trait loci (eQTLs) for the *AGTR2* gene in the uterus using data from the GTEx database [40] (Fig. 1 and Additional file 1, spreadsheet GTEx V7 eQTLs in uterus). Collectively, these data indicate that *AGTR2* is a likely gene target explaining the non-coding GWAS association.

We then used publicly available functional genomics datasets to prioritize the likely causal SNP(s) from the 35 SNPs identified in the previous analysis. The logic underlying this approach is the non-coding region harboring the causal SNP probably functions as an enhancer to regulate *AGTR2* gene expression, and thus will be more likely to be marked with genetic and/or epigenetic marks that are typically associated with enhancers. We collected functional genomic datasets from a range of sources, including ENCODE [28], Cistrome [32], PAZAR [33], Re-Map [34], and NIH Roadmap Epigenomics [35]. All datasets were indexed by their genomic coordinates, which were used to intersect with the LD expanded list of SNPs of interest. We prioritized SNPs based on their overlap with both likely regulatory regions (e.g., regions with histone marks such as H3K27ac) and TF ChIP-seq datasets (Fig. 1 and Additional file 1, spreadsheets ChIP-seq no-TF overlaps and ChIP-seq TF overlaps). Collectively, rs7889204 emerged as the most likely functional candidate SNP (Fig. 1 and Additional file 1, spreadsheet ChIP-seq TF-based diff binding). Subsequent functional analysis thus focused on this potential causal SNP. A flowchart is provided (Fig. 1) that documents the complete process — results and intermediate output can be found in Additional file 1.

Differential TF binding analysis for rs7889204 performed using the CisBP database [41] yielded three strong candidates, all of which are predicted to bind more strongly to the T allele: CEBPB, HOXA, and FOX family members. We selected FOXA1 for experimental validation because the SNP is inside FOXA1 ChIP-seq peaks in multiple experiments, and CEBPB and HOXA10 were selected based on known biological roles.

### rs7889204 is a tissue-specific eQTL for *AGTR2*

**Fig. 2:**
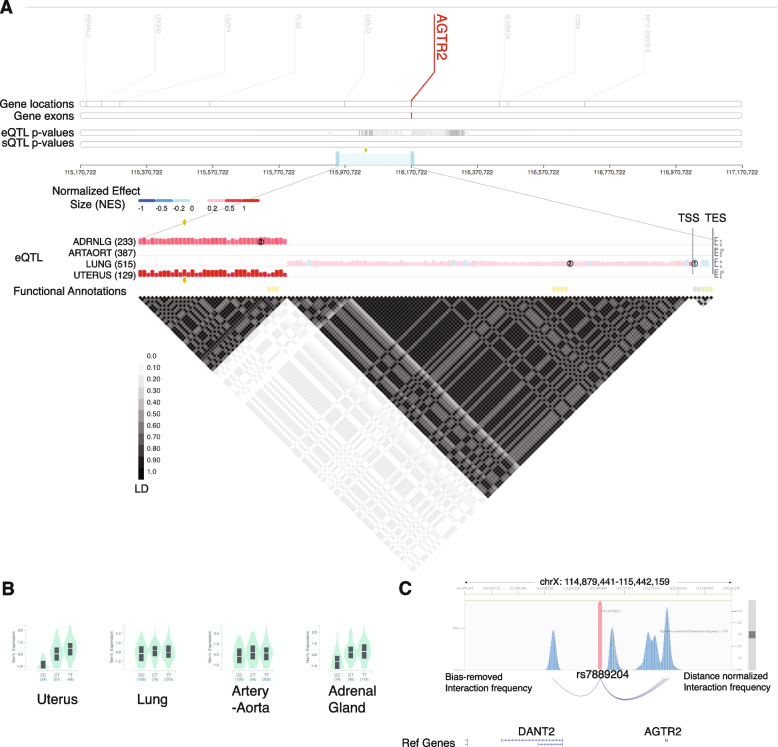
Rs7889204 likely acts by impacting *AGTR2* expression level. **A** Locus view of rs7889204. Red line indicates the location of the *AGTR2* gene on the X chromosome. The diamond in the zoomed view indicates the SNP rs7889204. **B** Violin plots demonstrating that rs7889204 is an eQTL for *AGTR2,* with the strongest effect in the uterus. **C** The rs7889204 locus physically interacts with the promoter region of *AGTR2*. Chromatin conformation analysis reveals a high distance normalized interaction frequency between the rs7889204 LD block (vertical red var) and the *AGTR2* promoter. Higher peaks indicate stronger interactions. Data displayed were obtained from human fibroblasts. Analysis was performed using the 3D Genome Interactive Viewer and Database

We next examined the effect of rs7889204 on *AGTR2* gene expression using the GTEx dataset (Fig. 2). Figure 2A depicts the *AGTR2* locus, showing the position of *AGTR2* gene (marked in red) and the candidate SNP (middle panel, indicated by a red arrow). A zoomed-in view is also provided showing the effect of this SNP on the expression of the *AGTR2* gene in different tissues including the adrenal gland, artery-aorta, lung, and uterus. This eQTL analysis suggests that the region harboring rs7889204 has the largest effect in uterine tissue, with higher *AGTR2* expression observed for the reference (non-risk) “T” allele and lower expression for the non-reference (risk) “C” allele (Fig. 2B).

### rs7889204 forms chromatin loops to the promoter region of *AGTR2*

We next examined if the region surrounding this SNP has the potential to form enhancer-promoter interaction with *AGTR2*, which is essential to regulate target gene expression. We used rs7889204 as the bait on the 3D Genome Interactive Viewer and Database [36] (3DIV) (http://kobic.kr/3div/). We used default settings on the Hi-C page except we set the threshold on distance normalized interaction frequency to be 2.00. This analysis revealed that the regions harboring rs7889204 do indeed physically interact with the *AGTR2* promoter in fibroblasts (Fig. 2C). Although experimental data do not currently exist for examining chromatin interactions between rs7889204 and *AGTR2* in the context of preterm/gestational duration, these results suggest that such enhancer-promoter interactions are plausible in disease-relevant cell types.

### Human *AGTR2* expression and transcription factors in uterine tissue

**Fig. 3:**
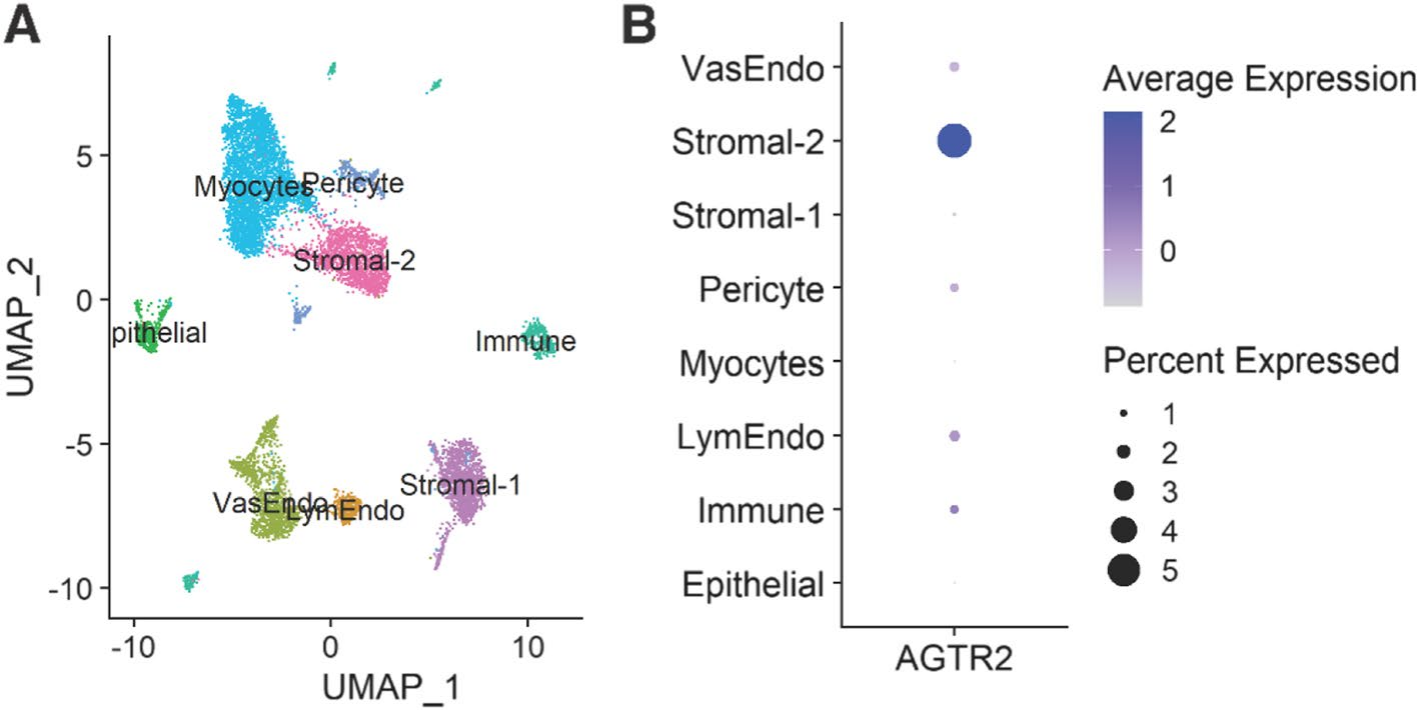
*AGTR2* expression in human uterus cells. **A** Uniform Manifold Approximation and Projection (UMAP) representation of 23,110 cells from 2 human uterine samples (PMID: 32,603,599. GSM4035471 and GSM4035470). Eight cellular populations were identified and labeled using different colors. **B** Dot plot showing *AGTR2* expression across 8 cell populations. *AGTR2* has low but highly selective and enriched expression in Stromal-2 cell populations (frequency: 5.2%, enrichment fold: 3.3, *P*-value: 1E − 05). Node size represents gene expression frequency in a cell type. Node color represents the average gene expression in a cell type

Being the most abundant cell type in the endometrium, endometrial stromal cells undergo a dramatic morphological and functional differentiation during decidualization and are essential for the establishment of a successful pregnancy [42]. Analysis of existing Hi-C data also suggests that such enhancer-promotor interactions are plausible in a fibroblast cell context (Fig. 2C). However, we were not able to detect endogenous *AGTR2* expression in the immortalized endometrium stromal fibroblast cell line (ATCC CRL-4003). It is also absent in the transcriptome study using this cell line before and after decidualization [31]. Gene expression profiling using microarray in the basal plate of the human maternal–fetal interface showed the expression of *AGTR2* is higher in the 37–40 week samples than in the 14–18 week samples (*P* = 0.01, BH-FDR = 0.09) [43], indicating the dynamic expression pattern of this gene. We expanded our search to single-cell transcriptomic studies, but *AGTR2* seems absent in the datasets we have checked [44–47]. E.g., re-analysis of single-cell data from the human early maternal–fetal interface showed *AGTR2* is only present in 10 cells out of 70,325 cells (Additional file 2). Our attention was then directed to lung research, in which tissue shows biased *AGTR2* expression. Re-analysis of single cell/single nuclei RNA-seq in human uterus tissue from this study [48] shows *AGTR2* has low but highly selective and enriched expression in Stromal-2 cell populations as shown in Fig. 3, though the biological context of this data-driven unbiased cell type classification needs further investigation.

The computational analysis suggests that the rs7889204 genomic locus might act as an enhancer to regulate *AGTR2* gene expression in the uterine tissue. It further suggests that altered binding of HOXA10, CEBPB, and/or FOXA1 might explain the observed allele-dependent expression of *AGTR2* in uterine tissue. Previous studies have shown that HOXA10 might play key roles in directing endometrial stromal cell differentiation and is key for normal implantation [49, 50]; CEBPB is expressed at implantation sites in human and non-human primates, where intense nuclear expression of CEBPB has been observed in decidual stromal cells [51]. No direct connection between FOXA1 and pregnancy has been previously reported. Previous transcriptomic studies in immortalized human endometrium stromal cells before and after induced decidualization show expression of both HOXA10 and CEBPB, but not FOXA1 [31]. This finding is also confirmed by the TaqMan gene expression assay by our group (Additional file 2).

In the following in vitro studies, we chose the immortalized endometrium stromal fibroblast cell line (ATCC CRL-4003) knowing the limitations of lack of detectable expression of *AGTR2*.

### EMSA confirms allele-dependent binding of CEBPB and HOXA10 at rs7889204

**Fig. 4:**
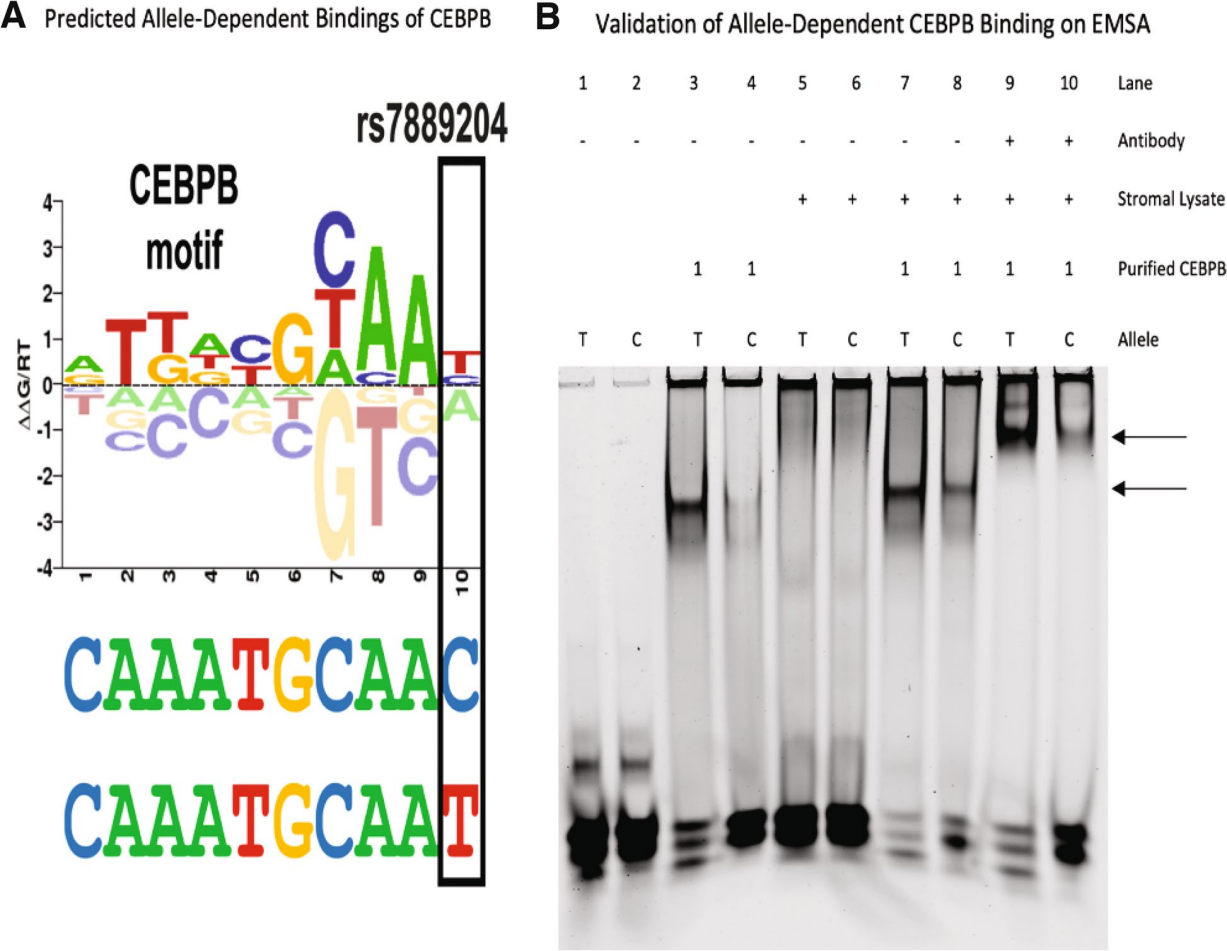
EMSA confirms allele-dependent binding of CEBPB at rs7889204. Panel **A** visually depicts the computational prediction that the rs7889204 T allele creates a stronger binding site for CCAAT Enhancer Binding Protein Beta (CEBPB). The sequence logo of the CEBPB binding motif (below) shows the DNA-binding preferences of CEBPB. Tall nucleotides above the dashed line indicate DNA bases that are preferred by CEBPB, whereas bases below the dashed line are disfavored. The *y*-axis indicates the relative free energies of binding for each nucleotide at each position. The height of each nucleotide can be interpreted as the free energy difference from the average (ΔΔG) in units of gas constant (R) and temperature (T). The sequence located in the *AGTR2* locus is shown directly below the *x*-axis, with the T allele for rs7889204 at the bottom. The T allele changes the sequence from C (most disfavored) to T (most preferred). Panel **B** shows the experimental validation of allele-dependent binding of CEBPB to rs7889204 using an electrophoretic mobility shift assay (EMSA). Arrows indicate allele-dependent binding of CEBPB (bottom arrow) and a “super shift” of the protein-DNA complex induced by the binding of DDK tagged antibody (CEBPB tagged with DDK motif) to the complex (top arrow)

Using an electrophoretic mobility shift assay (EMSA), we detected decreased binding of CEBPB to the risk “C” allele of rs7889204 as predicted using decidualized hESC (human endometrium stromal cell) nuclear lysate with recombinant CEBPB protein (Fig. 4). We also detected decreased binding of HOXA10 to the risk “C” allele of rs7889204 as predicted using decidualized hESC nuclear lysate with recombinant HOXA10 protein (Fig. S1). However, we did not detect substantial differences between the risk “C” and non-risk “T” alleles of rs7889204 using decidualized hESC nuclear lysate with recombinant FOXA1 protein (Fig. S2). Collectively, these results confirm the predictions of stronger binding of the HOXA10 and CEBPB transcription factors to the non-risk allele of rs7889204.

### The risk “C” allele of rs7889204 has decreased enhancer activity

**Fig. 5:**
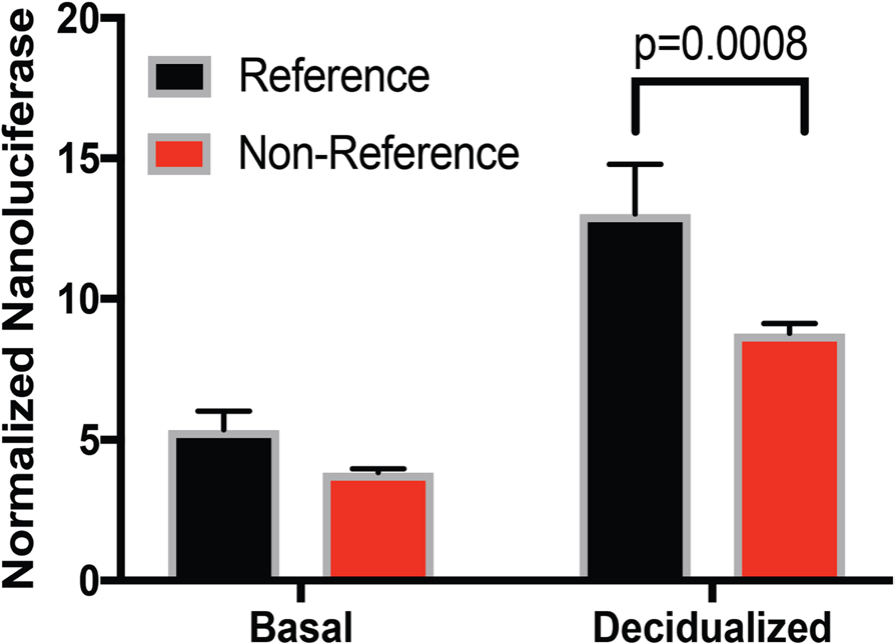
The risk “C” allele of rs7889204 shows decreased reporter activity. A 4.3-kb region surrounding rs7889204 with either the reference (T) or non-reference (C) SNP haplotypes were inserted in front of a 1 kb sequence including the first 816 bases before the transcription start site of *AGTR2* in a pNL1.1 nanoluciferase reporter construct. Nanoluciferase readings were normalized to the constitutive control for each replicate. Each bar represents the mean across three experimental replicates and the standard deviation

To determine whether the region surrounding rs7889204 possesses transcriptional enhancer activity and if the reference non-risk “T” and non-reference risk “C” alleles demonstrate differences in activity, we performed luciferase reporter experiments in both non-decidualized and decidualized hESC cells. The rs7889204 region and promoter region of *AGTR2* were placed in front of the nano-luciferase gene. Nano-luciferase expression was responsive to the *AGTR2* promoter both in non-decidualized and decidualized hESCs (Fig. 4); however, we observed greater expression in the *AGTR2* promoter-containing pNL1.1 vector after decidualization (Fig. 5). Importantly, the regulatory region containing the risk “C” allele for rs7889204 demonstrated significantly less nano-luciferase expression compared to the reference allele reporter construct. This decrease in nano-luciferase expression for the non-reference allele of rs7889204 was not strongly dependent on cellular conditions, as a significant, albeit slightly, weaker decrease was also observed in non-decidualized endometrial cells (Fig. 5). These results are consistent with the reduced transcription factor binding observed in EMSA (Fig. 4), collectively revealing a mechanism involving weaker CEBPB and HOXA10 binding to the rs7889204 risk allele, leading to reduced *AGTR2* expression.

## Discussion

**Fig. 6:**
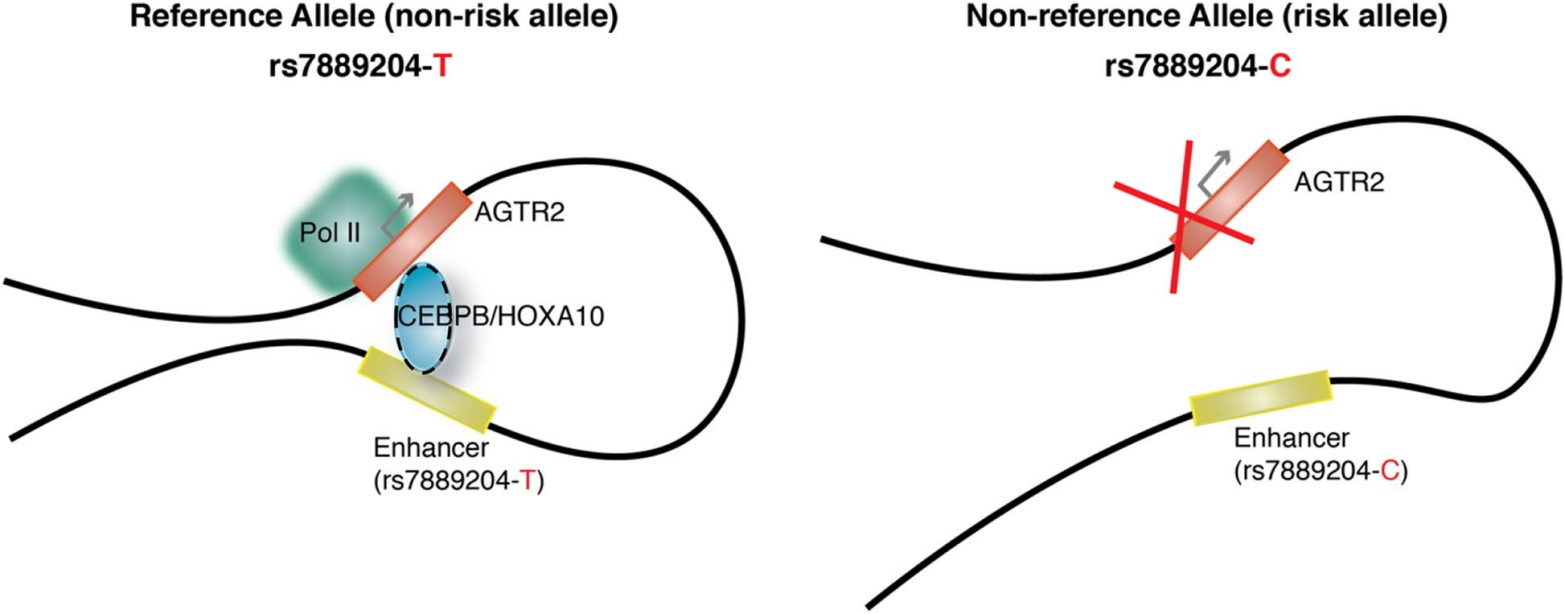
Schematic illustration of rs7889204’s function as an allele-dependent enhancer for *AGTR2*. The T allele of the non-coding variant rs7889204 results in stronger binding of the CEBPB and HOXA10 transcription factors, with subsequent activation of the target gene *AGTR2* in human endometrium stromal fibroblast cells. The C allele disrupts this interaction, resulting in down-regulation of the *AGTR2* target gene

Preterm birth remains a significant healthcare burden on a global scale due to a lack of progress in mechanistic insights to develop preventative measures. Using bioinformatics and functional analyses, we identified a functional mechanism in which rs7889204 at the *AGTR2* locus serves as part of a likely enhancer where the risk “C” allele shows decreased *AGTR2* expression potentially via allele-dependent binding of the transcription factors *CEBPB* and *HOXA10* (Fig. 6). The absence of evidence for the co-expression between *AGTR2* and *CEBPB* and *HOXA10* regulators is indicated by the dashed line, although it is still the prevailing scenario for such regulation.

In this study, we prioritized SNPs by interrogating available data at the genomic level. We carefully designed our prioritization procedure using existing genomic and epigenomic data sets, which successfully prioritized rs7889204 as the top causal genetic variant. We further demonstrated that rs7889204 can regulate *AGTR2* expression via the alteration of the activity of a likely enhancer, through allelic differential binding of the biologically relevant transcription factors *CEBPB* and *HOXA10*.

Our study has shown that the risk “C” allele of rs7889204 exhibits decreased transcription factor binding and enhancer activity in vitro. To confirm whether this finding leads to decreased *AGTR2* expression in vivo, further studies are necessary. Unfortunately, endogenous *AGTR2* expression was not detected in the cell lines used in this study (Additional file 2). Transcriptomic profiling using primary human endometrium biopsies in the proliferative phase also did not detect *AGTR2* expression [16]. To eliminate cell type heterogeneity effects, we also explored two publicly available single-cell transcriptomics data sets, with one focusing on the human midluteal implantation window [44] and the other on human endometrium during the menstrual cycle [47]. Unfortunately, neither dataset showed reliable *AGTR2* expression (Additional file 2).

Previous studies have reported that *AGTR2* expression is specific to defined tissues during development including the endometrium, uterus, and lung [13, 14]. Western blotting and PCR analysis have suggested its possible presence in myometrium and uncertain expression in the human placenta and endometrium [12, 49]. Our single-cell RNA-seq analysis supports the expression of *AGTR2* specifically in the endometrial stromal cell during pregnancy (Fig. 3) which is consistent with the likely cell context of effect for *Wnt4* in modulation of gestational duration which we have previously described [8]. The expression of *AGTR2* declines during pregnancy, with negligible levels detected in late pregnancy [12]. These findings suggest that *AGTR2* expression is regulated spatially and temporally during pregnancy and further complicates its detection in human pregnancy though also lends credence to the notion that *AGTR2* promotes gestational prolongation.

The restricted ability to detect *AGTR2* expression throughout pregnancy is a limitation in our findings. While the absence of expression in the cell line we used is a limitation in interpretation, like many human pregnancy-related expression changes, we are only beginning to understand the context for their expression. These limitations highlight the importance of understanding tissue-specific regulation of *AGTR2* expression and the need for further studies in animal models to elucidate the mechanism of *AGTR2* regulation during pregnancy. The induction of specific primate genes in the context of decidual-placental interaction has been a challenging task despite providing all necessary signals. For instance, the presence of the human corticotropin-releasing hormone (*CRH*) has been well-documented in placental syncytiotrophoblast [52]. However, even in primary syncytialized trophoblasts, endogenous expression of *CRH* has not been detected. Instead, researchers had to create a novel mouse model by incorporating a bacterial artificial chromosome (BAC) with the human *CRH* gene and approximately 180 kb of flanking sequence into the genome of FVB/N mice [53], which showed placental expression of *CRH*. In the case of *AGTR2*, we believe the endometrial stromal cell line provides the permissive environment to allow the EMSA and luciferase studies we have performed to reveal mechanistic insights regarding rs7889204. Follow-up studies in animals will be the most meaningful next step for the future.

To gain a deeper understanding of the mechanism underlying *AGTR2*’s regulation of birth timing, additional research using animal models is a logical next step. Specifically, it is important to characterize *AGTR2* expression throughout gestation in nonhuman primates and/or in *AGTR2* BAC transgenic and gene-edited mice. The use of animal models will allow for the investigation of *AGTR2* regulation at both the molecular and physiological levels, further validating the causal relationship between the non-coding SNP rs7889204 and PTB. New techniques in profiling multiple layers of genetic and epigenetic marks simultaneously [54] will also facilitate the identification of regulatory mechanisms and lead to better understanding of the interplay between genetic factors in preterm birth. Such studies could provide valuable insights into the complex interplay of factors involved in the timing of birth and may have important implications for the development of strategies to prevent preterm birth.

The potential for *AGTR2* as a target for drug development has been demonstrated by the development of a number of agonists and antagonists. Our results further underscore the potential for modulation of *AGTR2* and its regulatory transcription factors, *CEBPB* and *HOXA10*, as promising targets for the prevention and treatment of preterm birth.

## Conclusions

This study is the first single locus post-GWAS functional study in preterm birth. Using a combination of experimental and computational tools, we linked a non-coding variant in the human genome to its target gene, *AGTR2*. Our results also will draw more attention to the study of *AGTR2*’s role in preterm birth. As a druggable target, our results will greatly assist the utility of *AGTR2* modulation and its regulators for preterm birth prevention and therapy. This work will be of broad interest to the scientific community and spanning across many disciplines and especially in the areas of post-GWAS functional studies.

## Supplementary Information

**Additional file 1.** Prioritization of functional SNPs and predication of allele-dependent TF binding

**Additional file 2.** Human AGTR2 expression analysis.

**Additional file 3: Fig. S1.** EMSA confirms allele-dependent binding of HOXA10 at rs7889204. **Fig. S2. **EMSA does not confirm allele-dependent binding of FOXA1 at rs7889204.

## Data Availability

All data needed to evaluate the conclusions in the paper are present in the paper and/or the Additional Materials.

## Abbreviations

AGTR2: Angiotensin II receptor type 2
CEBPB: CCAAT enhancer binding protein beta
CRH: Corticotropin-releasing hormone
DAPA: DNA affinity precipitation assays
EMSA: Electromobility shift assays
eQTL: Expression quantitative trait loci
ESC: Embryonic stem cells
FOXA1: Forkhead box protein A1
GTEx: Genotype-Tissue Expression
GWAS: Genome-wide association studies
hESF: Human endometrium stromal fibroblast cell
HOXA10: Homeobox A10
LD: Linkage disequilibrium
PTB: Preterm birth
SNPs: Single nucleotide polymorphisms
TAD: Topologically associated chromatin domains
TF: Transcription factor
TSS: Transcription start site
