# Supplementary material for: A functional mechanism for a non-coding variant near AGTR2 associated with risk for preterm birth
Source: BMC Med. 2023 Jul 17;21:258. doi: 10.1186/s12916-023-02973-w (PMC10351137; doi:10.1186/s12916-023-02973-w)
Supplement: Supplementary file 3 — Additional file 3: Fig. S1. EMSA confirms allele-dependent binding of HOXA10 at rs7889204. Fig. S2. EMSA does not confirm allele-dependent binding of FOXA1 at rs7889204. [file 12916_2023_2973_MOESM3_ESM.pdf]

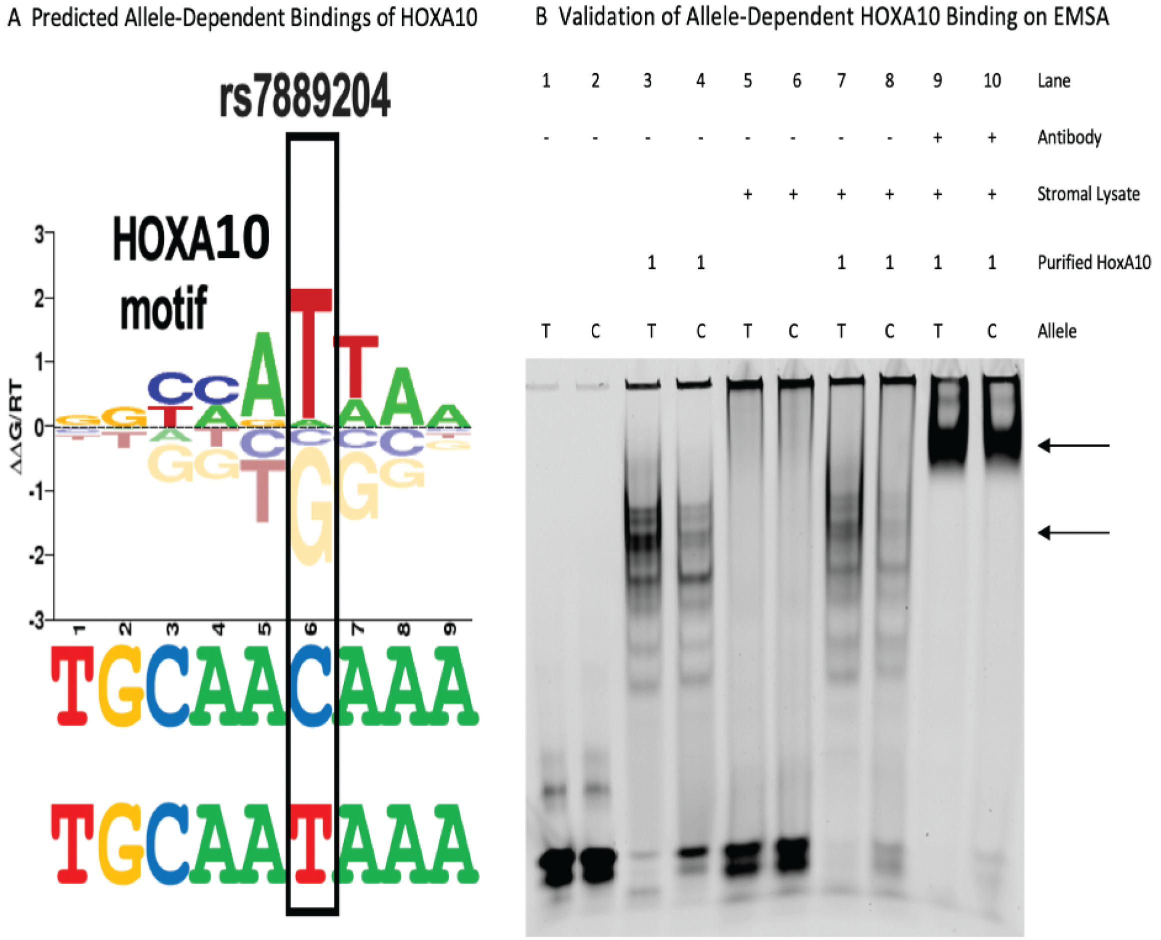

Fig. S1. EMSA confirms allele-dependent binding of HOXA10 at rs7889204. Panel A visually depicts the computational prediction that the rs7889204 T allele creates a stronger binding site for HOXA10. The sequence logo of the HOXA10 binding motif (below) shows the DNA-binding preferences of HOXA10. Tall nucleotides above the dashed line indicate DNA bases that are preferred by HOXA10, whereas bases below the dashed line are disfavored. The y axis indicates the relative free energies of binding for each nucleotide at each position. The height of each nucleotide can be interpreted as the free energy difference from the average (ΔΔG) in units of gas constant (R) and temperature (T). The sequence located in the *AGTR2* locus is shown directly below the x axis, with the T allele for rs7889204 at the bottom. The T allele changes the sequence from C (most disfavored) to T (most preferred). Panel B shows the experimental validation of allele-dependent binding of HOXA10 to rs7889204 using an electrophoretic mobility shift assay (EMSA). Arrows indicate allele-dependent binding of HOXA10 (bottom arrow) and a “super shift” of the protein-DNA complex induced by the binding of DDK tagged antibody (HOXA10 tagged with DDK motif) to the complex (top arrow).

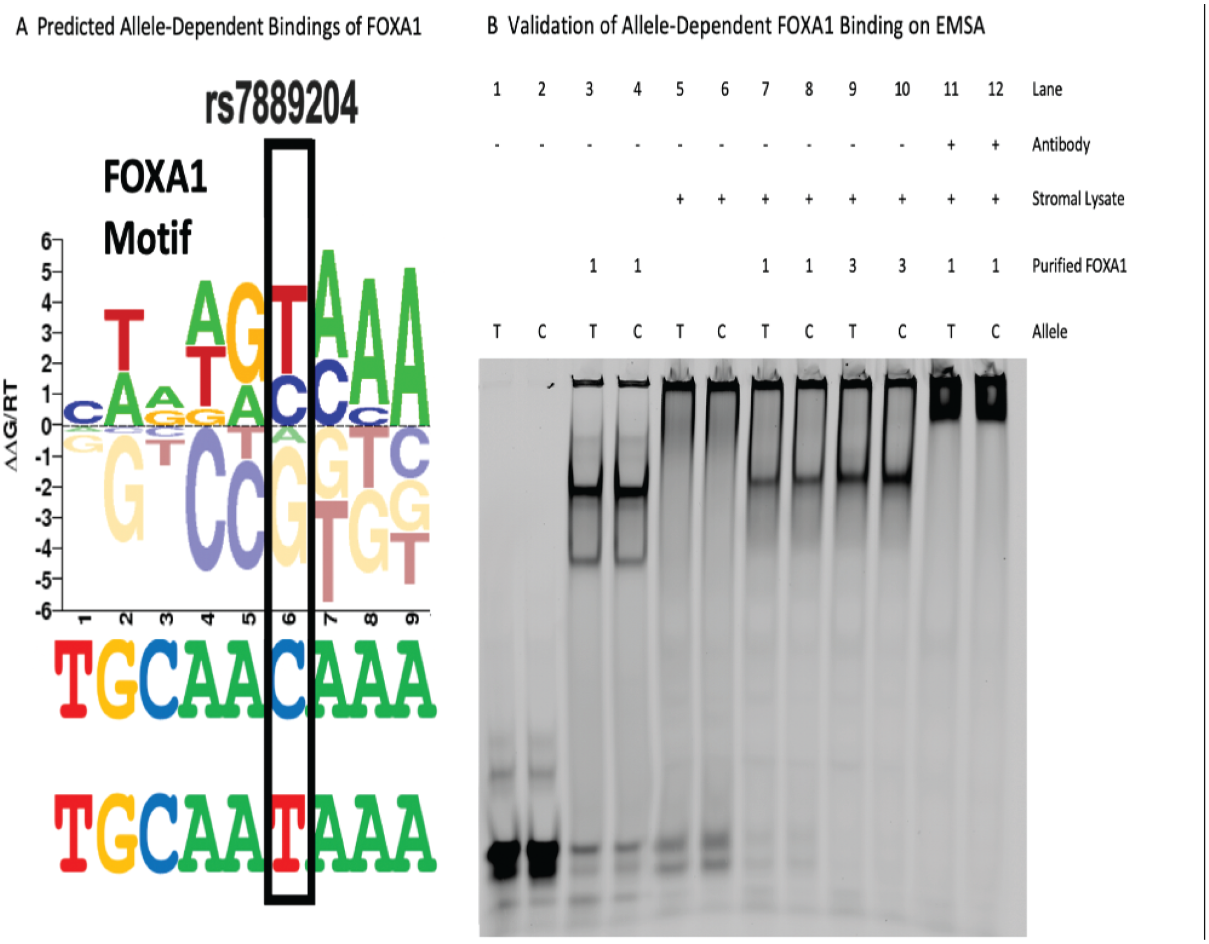

Fig. S2. EMSA does not confirm allele-dependent binding of FOXA1 at rs7889204. Panel A visually depicts the computational prediction that the rs7889204 T allele creates a stronger binding site for FOXA1. The sequence logo of the FOXA1 binding motif (below) shows the DNA-binding preferences of FOXA1. Tall nucleotides above the dashed line indicate DNA bases that are preferred by FOXA1, whereas bases below the dashed line are disfavored. The y axis indicates the relative free energies of binding for each nucleotide at each position. The height of each nucleotide can be interpreted as the free energy difference from the average (ΔΔG) in units of gas constant (R) and temperature (T). The sequence located in the *AGTR2* locus is shown directly below the x axis, with the T allele for rs7889204 at the bottom. The T allele changes the sequence from C (relatively disfavored) to T (most preferred). Panel B shows the attempted experimental validation of allele-dependent binding of FOXA1 to rs7889204 using an electrophoretic mobility shift assay (EMSA). Relatively equal binding of FOXA1 is observed between the two alleles, which is also observed subsequent to “supershift” using a DDK tagged antibody (FOXA1 tagged with DDK motif) to the complex.

.
